# Clinical characteristics, risk factors and diagnostic outcomes of patients presented with indeterminate biliary stricture: A multicenter study

**DOI:** 10.3389/fmed.2022.1018201

**Published:** 2023-01-09

**Authors:** Mohammed Tag-Adeen, Mohamed Malak, Muhammad Abdel-Gawad, Ahmed Abu-Elfatth, Ramadan H. Eldamarawy, Ahmed Alzamzamy, Mohamed Elbasiony, Ramy M. Elsharkawy, Fathiya El-Raey, Ahmed N. Basiony, Ahmed Qasem, Zakarya Shady, Ahmed S. Abdelmohsen, Doaa Abdeltawab, Mahmoud Farouk, Ola M. Fouad, Ahmed Rabie, Abdul-Hakim Erian, Ahlam Sapra, Wael Shaibat-Alhamd, Ashraf Aboubakr, Dalia Omran, Mohamed Alboraie

**Affiliations:** ^1^Division of Gastroenterology and Hepatology, Department of Internal Medicine, Qena Faculty of Medicine, South Valley University, Qena, Egypt; ^2^Division of Gastroenterology and Hepatology, Department of Internal Medicine, Faculty of Medicine, Sohag University, Sohag, Egypt; ^3^Hepatology, Gastroenterology, and Infectious Diseases, Al-Azhar University, Assiut, Egypt; ^4^Department of Tropical Medicine and Gastroenterology, Assuit University, Assiut, Egypt; ^5^Department of Internal Medicine, Al-Azhar University, Cairo, Egypt; ^6^Department of Gastroenterology and Heptology, Maadi Armed Forces Medical Complex, Military Medical Academy, Cairo, Egypt; ^7^Division of Gastroenterology and Hepatology, Department of Internal Medicine, Faculty of Medicine, Mansoura University, Mansoura, Dakahlya, Egypt; ^8^Department of Tropical Medicine and Gastroenterology, Faculty of Medicine, Sohag University, Sohag, Egypt; ^9^Department of Hepatogastroenterology and Infectious Diseases, Al-Azhar University, Damietta, Egypt; ^10^Department of Tropical Medicine, Ain Shams University, Cairo, Egypt; ^11^Department of Pathology, Faculty of Medicine, Al-Azhar University, Damietta, Egypt; ^12^Division of Gastroenterology and Hepatology, Department of Internal Medicine, Al-Azhar Faculty of Medicine, Al-Azhar University, Assiut, Egypt; ^13^Department of Endemic Medicine and Hepatology, Faculty of Medicine, Cairo University, Giza, Egypt

**Keywords:** cholangiocarcinoma (CCA), biliary stricture, ERCP, MRI, MRCP, CECT

## Abstract

**Background and aim:**

Indeterminate biliary stricture (IBS) is a frequently encountered clinical problem. In this study, we aimed to highlight the clinical characteristics, risk factors and diagnostic outcomes of patients presented with indeterminate biliary stricture.

**Method:**

A Retrospective multicenter study included all patients diagnosed with IBS in the participating centers between 2017 and 2021. Data regarding IBS such as presentations, patient characteristics, diagnostic and therapeutic modalities were collected from the patients' records and then were analyzed.

**Results:**

Data of 315 patients with IBS were retrospectively collected from 7 medical centers with mean age: 62.6 ± 11 years, females: 40.3% and smokers: 44.8%. For diagnosing stricture; Magnetic resonance imaging/Magnetic resonance cholangiopancreatography (MRI/MRCP) was the most frequently requested imaging modality in all patients, Contrast enhanced computerized tomography (CECT) in 85% and endoscopic ultrasound (EUS) in 23.8%. Tissue diagnosis of cholangiocarcinoma was achieved in 14% only. The used therapeutic modalities were endoscopic retrograde cholangiopancreatography (ERCP)/stenting in 70.5%, percutaneous trans-hepatic biliary drainage (PTD): 17.8%, EUS guided drainage: 0.3%, and surgical resection in 8%. The most frequent type of strictures was distal stricture in 181 patients, perihilar in 128 and intrahepatic in 6. Distal strictures had significant male predominance, with higher role for EUS for diagnosis and higher role for ERCP/stenting for drainage, while in the perihilar strictures, there was higher role for CECT and MRI/MRCP for diagnosis and more frequent use of PTD for drainage.

**Conclusion:**

Indeterminate biliary stricture is a challenging clinical problem with lack of tissue diagnosis in most of cases mandates an urgent consensus diagnostic and treatment guidelines.

## Introduction

The indeterminate biliary stricture (IBS) is a term that could be used referring to stricture in the biliary tree with unidentified etiology after completing initial work up including transabdominal ultrasound (US), computerized tomography (CT), magnetic resonance imaging (MRI) and endoscopic retrograde cholangiopancreatography (ERCP) with transpapillary biopsy and/or standard cytologic brushing (1, 2).

The differential diagnosis of this term may include benign conditions such as iatrogenic post-surgical biliary strictures, primary sclerosing cholangitis (PSC), IgG4-related sclerosing cholangitis, recurrent pyogenic cholangitis, ischemic cholangiopathy, AIDS-associated cholangiopathy, acute or chronic pancreatitis, autoimmune pancreatitis, or malignant conditions such as cholangiocarcinoma (CCA) or pancreatic cancer (3–5).

CCA is the primary tumor of the bile ducts that accounts for about 15% of all primary liver tumors and comes second to hepatocellular carcinoma (HCC) regarding its frequency. Also; it represents ~3% of all gastrointestinal cancers and forms a global health problem with an increasing incidence worldwide in the past few decades (6–8). According to the anatomical location, CCA is classified to distal CCA when it is limited to the common bile duct (CBD) distal to the cystic duct (CD) insertion and without extension/invasion of the papilla of Vater (PV); perihilar CCA when it involves the common hepatic duct CHD, and/or right hepatic duct (RHD), and/or left hepatic duct (LHD); and intrahepatic CCA when the involvement is limited only to the intrahepatic biliary radicals (6, 7, 9). Diagnosis of CCA is usually suspected based on radiologic findings of biliary stricture or mass-forming stricture in the setting of assessment of patient presented with cholestatic jaundice, and is then confirmed through pathologic examination of obtained specimen either by endoscopic ultrasound fine needle aspiration/biopsy (EUS-FNA/B), cholangioscopy (CS) or transcutaneous rout (10). Unfortunately; despite the recent improvement in the diagnosis and treatment of CCA, patient prognosis has not been improved substantially with disappointing 5-year survival and tumor recurrence rates after resection (11–13).

## Aim

To highlight the clinical characteristics, risk factors and diagnostic outcomes of patients presented with indeterminate biliary stricture.

## Methods

A Retrospective multicenter study included all patients diagnosed with biliary strictures with no identified cause after initial diagnostic work up between 2017 and 2021. Data regarding IBS such as clinical presentations, patient characteristics, relation to common hepatobiliary pathogens, available diagnostic tools, and treatment modalities were collected from the patients' records and then were analyzed. Correlation between the anatomical site of stricture; distal, perihilar or intrahepatic, and risk factors were also studied. The included cases were classified to distal IBS when the stricture was limited to the CBD distal to the CD insertion without involving the PV; perihilar IBS when involved the CHD, and/or RHD, and/or LHD; and intrahepatic stricture when the involvement was limited only to the intrahepatic biliary radicals.

### Exclusion criteria

Patients with history and/or imaging findings suggestive of iatrogenic biliary stricture, pyogenic cholangitis, PSC, pancreatitis, primary pancreatic or gallbladder neoplastic lesions were excluded.

### Study definitions

All radiographic descriptive terms like stricture or mass-forming stricture involving the biliary tree without well-identified etiology after initial imaging including (US, CT and/or MRI/MRCP) were defined as indeterminate biliary stricture (IBS) in this study.

### Statistical analysis

Data were collected from the participating centers in a single predesigned excel sheet and then analyzed using Statistical Package for the Social Science (SPSS version 20, IBM and Armonk, NY, USA). Continuous variables were expressed in form of mean ± standard deviation (SD) or median and range. Nominal variables were expressed as frequency and percentage. Chi squared test was used to compare nominal data and ANOVA to compare continuous data. Level of confidence was kept at 95% and *p*-value < 0.05 was significant.

## Results

This study has included 7 medical centers from Egypt; Qena University Hospital, Qena; Sohag University Hospital, Sohag; Assiut University Hospital, Assiut; Al-Azhar University Hospital, Assiut; Al-Azhar University Hospital, Cairo; Maadi Armed Forces Medical Complex, Military Medical Academy, Cairo; Mansoura University Hospital, Dakahlya.

A Total of 315 patients with IBS were enrolled, the mean age was 62.6 ± 11 years, females were 127 (40.3%), smokers were 141 (44.8%). Personal history of malignancy was positive in 18 (5.7%) of patients and 12 patients (3.8%) had a positive family history for malignant tumors, Table 1 shows the rest of demographic criteria of the included patients.

**Table 1:** Demographic data of the included patients; data are presented as numbers and percent.

**Variables**	***n* = 315 patients**
Age (years, mean ± SD)	62.67 ± 11.36
Females	127 (40.3%)
Smokers	141 (44.8%)
Urban	77 (24.4%)
**Comorbidities**	
HCV	75 (23.8%)
HBV	5 (1.6%)
DM	127 (40.3%)
HTN	69 (21.9%)
IHD	16 (5.1%)
Choledochal cyst	0
Obesity	6 (1.9%)
Liver cirrhosis	17 (5.4%)
Heart failure	1 (0.3%)
Gallstones^*^	83 (26.3%)
**Other cancers**	
Breast cancer	1 (0.3%)
Hepatocellular carcinoma	11 (3.4%)
Colorectal cancer	3 (0.9%)
Gastric cancer	1 (0.3%)
Prostatic cancer	1 (0.3%)
Lung cancer	1 (0.3%)
Family history of cancers	12 (3.8%)

* Descriptive terms like thick wall or distended gallbladder without visible stones, surgically removed gallbladder, or unavailable data about the gallbladder status, all were considered as no gallstones in our analysis.

Obstructive jaundice work-up was the reason for hospital admission in 248 patients (79%) with median serum bilirubin: 12 mg/dl (range: 0.3–29.7), median ALP: 448 IU/L (range: 56–3,220), median CEA: 12 ng/ml (range: 0.05–1,224) and median CA19-9: 93 U/ml (range: 5.50–943), Table 2 shows the biochemical profile of the included patients.

**Table 2:** Laboratory data of the included patients (*n* = 315); data are expressed as median (range) and mean ± standard deviation (SD).

**Variables (normal ranges)**	**Median (range)**	**Mean ±SD**
Creatinine (0.4–1.4) mg/dl	1.10 (0.21–5)	
INR (1–1.2)	1.20 (0.80–3.50)	
Bilirubin (up to 1) mg/dl	12 (0.30–29.77)	
Direct Bilirubin (up to 0.3) mg/dl	10 (0.12–26.50)	
ALT (up to 40) IU/L	70 (6–790)	
AST (up to 40) IU/L	77 (9–624)	
ALP (40–130) IU/L	448 (56–3220)	
Hemoglobin (12–16) g/dl	11.15 (5.10–16.40)	11.19 ± 1.94
WBCs (4–10 × 103)	9 (1–48)	
Platelets (150–350 × 103)	215 (12.80–713)	
Amylase (30–110) U/L	73.5 (12–980)	
CEA (0–2.5) ng/mL	12 (0.05–1224)	
CA 19–9 (0–37) U/mL	93 (5.50–943)	

The most frequently requested imaging modality for initial assessment of IBS was MRI/MRCP in all included patients, followed by CECT in 268 patients (85%) and EUS in 75 patients (23.8%). Definite tissue-based diagnosis of cholangiocarcinoma was achieved only in 44 cases (14%) of the included IBS cases while no definite diagnosis was achieved in the rest of cases 271 (86%).

ERCP with CBD stent insertion was the commonly used therapeutic modality in 222 patients (70.5%), followed by percutaneous trans-hepatic drainage (PTD) in 56 (17.8%) and EUS guided drainage only in 1 patient (0.3%). On the other hand; 25 patients (8%) were subjected to surgical resection without prior biliary drainage. While 12 patients (3.8%) of cases had no available data regarding the palliative treatment they received.

**Figure 1:**
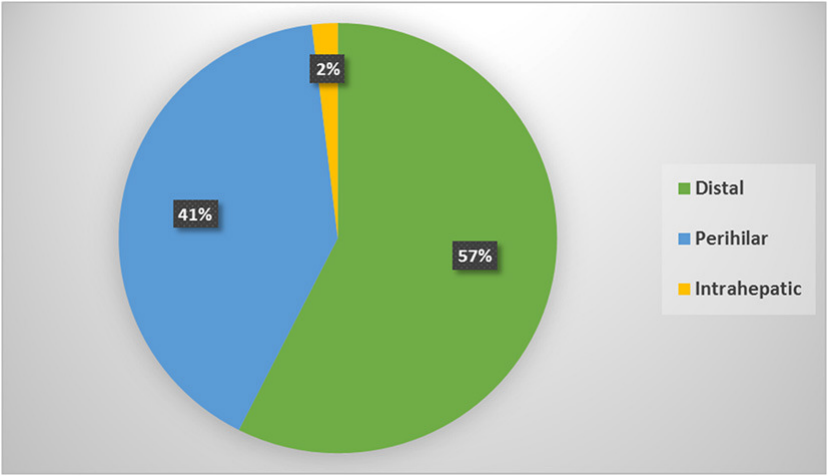
Percent of different types of biliary strictures (BS) according to our results.

According to its anatomical location; the most frequent type was distal IBS in 181 patents, perihilar in 128 and intrahepatic in 6 patients only; Figure 1. Comparison among different types showed statistically significant male predominance (*p* = 0.01) in the distal strictures with higher role for EUS (*p* < 0.0001) for diagnosis and higher role for ERCP/stenting for drainage. While, in the perihilar strictures, there was higher role for CECT (*p* = 0.02) and MRI/MRCP (*p* < 0.0001) for diagnosis and frequent use of PTD for drainage (*p* = 0.03). One out of 6 patients with intrahepatic strictures was positive for HBV infection (*p* < 0.0001). The rest of variables showed statistically insignificant distribution among different CCA types, Table 3.

**Table 3:** Distribution of different variables according to the anatomical location of biliary stricture (*n* = 315).

**Variables**		***N*** = **315**			***p*-value**
		**Distal (*****n*** = **181)**	**Perihilar (*****n*** = **128)**	**Intrahepatic** **(*****n*** = **6)**	
Age (years; mean ± SD)		63.84 ± 11.27	61.19 ± 10.67	59.17 ± 22.49	0.09
Gender	Male	119 (65.7%)	65 (50.8%)	4 (66.7%)	0.02
	Female	62 (34.3%)	63 (49.2%)	2 (33.3%)	
Smoking		85 (47%)	52 (40.6%)	4 (66.7%)	0.30
Diabetes		70 (38.7%)	55 (43%)	2 (33.3%)	0.70
Gallstones		49 (27.1%)	32 (25%)	2 (33.3%)	0.85
HCV infection		46 (25.7%)	28 (22.4%)	1 (16.7%)	0.73
HBV infection		2 (1.1%)	2 (1.6%)	1 (16.7%)	0.01
Family history		4 (2.2%)	8 (6.3%)	0	0.16
Final diagnostic tool	CECT/MRI/EUS	170 (93.9%)	97 (75.8%)	4 (66.7%)	0.001
	Tissue	11 (6.1%)	31 (24.2%)	2 (33.3%)	
Therapeutic intervention (biliary drainage or surgical resection)	ERCP stenting (*N* = 222, 70%)	123 (68%)	94 (73.4%)	5 (83.3%)	
	PTD (*N* = 56, 17.8%)	32 (17.7%)	23 (18%)	1 (16.7%)	0.93
	EUS-BD (*N* = 1, 0.3%)	1 (0.6%)	0	0	
	Surgical resection (*N* = 25. 7.9%)	18 (9.4%)	7 (5.5%)	0	
	Not available (*N* = 11, 3.5%)	7 (3.9%)	4 (3.1%)	0	

Data expressed as frequency (percentage) unless indicated; (p-value < 0.05).

## Discussion

Meticulous multimodality diagnostic work-up including CA19-9, CEA, contrast enhanced-computerized tomography (CECT), magnetic resonance imaging/magnetic resonance cholangiopancreatography (MRI/MRCP), EUS/EUS-FNA, brush cytology and CS is usually required to distinguish benign from malignant biliary strictures (14).

As imaging plays a crucial role in the diagnostic work-up of IBS, certain radiologic findings are suggestive of malignancy including duct hyperenhancement, ductal wall thickening, irregularity in the shape of the biliary stricture, regional lymph node enlargement, or a mass lesion (15). However; many factors can influence the diagnostic accuracy and staging capabilities of the different imaging modalities such as the anatomical location of the stricture; distal, perihilar or intrahepatic and its growth patterns; stricture, mass-forming stricture or infiltrative lesion (16, 17).

Multi-slice CECT is considered the standard imaging modality for the preoperative assessment of both intrahepatic and perihilar CCA; it provides a comprehensive locoregional evaluation of the primary lesion, the relationship with adjacent structures, vascular invasion and potential abdominal and/or extra-abdominal spread (18). Similarly; MRI is an accurate method for diagnosis and staging of CCA, with additional benefit of using specific sequences like diffusion-weighted imaging and MRCP which plays a critical role in perihilar CCA staging (19).

It is worthy mention that endoscopic ultrasound fine needle aspiration (EUS-FNA) has an important role in the diagnosis of biliary stricture with a sensitivity of 80% and a specificity of 97% for malignancy detection (20, 21). Also; the sensitivity and specificity for the diagnosis of CCA may be improved to 90% when taking biopsies of a suspicious stricture by direct cholangioscopy (21).

Our result showed that the most frequently requested imaging modality for assessing IBS was MRI/MRCP almost in all included patients followed by CECT in 85%. The superiority of using MRI/MRCP over CETC assumed to the need for contrast injection in CT which may be not appropriate in patients with impaired kidney function and patients' preference, otherwise; no institutional policy recommended the use of MRI/MRCP over CECT in the participating centers. EUS was used in 23.8% of patients only which elucidated a crucial role of both MRI/MRCP and CECT for assessing biliary strictures in the included centers compared to EUS, EUS-FNA and CS. That may be attributed to unavailability of EUS, EUS experts and CS in most of centers, as well as the added costs of these procedures.

CCA at its early stage is usually asymptomatic disease while in the late stages obstructive jaundice is the most frequent presenting symptom particularly for distal and perihilar CCA, and with exclusion of pancreatic cancer, CCA represents the main cause of neoplastic-featuring biliary strictures (22). In this study, tissue-confirmed diagnosis of CCA was achieved only in 44 (14%) in the included cases which sheds light on the major diagnostic problem of IBS in the included centers. Unfortunately, most of the included patients had jaundice as a presenting problem of their disease which indicates advanced disease at the onset of diagnosis.

Despite ERCP provides the best palliative management in jaundiced patients with surgically unresectable lesions, it is not currently recommended in potentially resectable lesions unless the patient is not surgical candidate. Beside its crucial therapeutic role, ERCP has an important diagnostic value by determining the location and extent of the biliary stricture, and providing tissue using cytology brush or fluoroscopic biopsy. Some morphologic criteria may suggest the malignant nature of IBS such as presence of long, irregular strictures with shelf-like morphology, and accompanied dilatation of the pancreatic duct (double duct sign) (19, 23). On the other hand, although ERCP-obtained-biliary samples provides high specificity of 95% for diagnosing malignancy, its sensitivity is low, about 23–56% for brushing cytology and 33–65% for fluoroscopic biopsies (24, 25). Considering the previously mentioned fact of low sensitivity as well as the limitations of using either techniques in some lesions particularly hilar ones, the included centers and expertises mostly confined brushing cytology and fluoroscopic biopsies to research purposes rather than doing it on routine basis. Additional advantage of ERCP in assessment of BS could be achieved by doing intraductal endoscopic ultrasound (IDUS) which provides real-time cross-sectional images of the bile ducts and periductal structures. IDUS criteria suggestive for malignancy include disruption of layers, eccentric wall thickening, enlarged lymph nodes, and hypoechoic sessile masses with signs of tissue or vascular invasion (26, 27). In a previous large retrospective study, the sensitivity, specificity and accuracy of IDUS were 93.2, 89.5, and 91.4% respectively in the evaluation of cancers of the bile duct, pancreas, ampullary, and gallbladder (28).

To our knowledge, this is the first work that studied reasonable number of patients presented with IBS to reflect its current insight in Egypt. Also, in the spotlight of this study, and in order to improve the diagnostic outcome of IBS, several modalities and technologies should be added to the diagnostic armamentarium of IBS such as single operator CS (29, 30), intraductal ultrasonography (28, 31), confocal laser endomicroscopy (CLE) (32–36), fluorescent *in situ* hybridization (FISH) (37–39), bile duct fluid biomarkers (40, 41), and digital image analysis (42). However; the study had some limitations including being retrospective, limited number of patients with the final diagnosis achieved, lack of long-term follow up, and missing important data such as the definite treatment, surgical resectability and overall outcome.

## Conclusion

Indeterminate biliary strictures are a frequently encountered, challenging problem in clinical practice. Distal strictures are more frequent in males with higher role for EUS for its diagnosis. ERCP/stenting is the commonest available palliative treatment with frequent need for PTD in the perihilar type. Tissue-based diagnosis of cholangiocarcinoma is available only for limited number of cases which indicate an urgent need for consensus diagnostic and management criteria/guidelines for indeterminate biliary strictures.

## Data availability statement

The raw data supporting the conclusions of this article will be made available by the authors, without undue reservation.

## Ethics statement

The study was reviewed and approved by the institutional review board of Faculty of Medicine, Al-Azhar University, Damietta, Egypt. Patients were not required to give informed consent to this study because the analysis used anonymous clinical data that were obtained from the medical records of the participating centers.

## Author contributions

MK designed the study idea, assisted with data collection and manuscript drafting and journal submission. MM and DA assisted with data collection and manuscript drafting. MA-G and AA-E assisted with data collection and statistical analysis. FE-R and AR obtained the ethical committee approval and participated in manuscript writing. AAl, AB, OF, AQ, AAbd, MF, REld, ZS, ME, REls, and WS-A participated in designing the methodology, and data collection and analysis. A-HE, AS, AAbo, DO, and MA designed and oversighted the study and revised the final edit of the manuscript and prepared it for submission. All authors read and approved the final manuscript.

## References

[1] Sethi A Howell DA. The indeterminate biliary stricture. In:Chandrasekhara V Elmunzer BJ Khashab MA, editors. Clinical Gastrointestinal Endoscopy. 3rd Edition. Philadelphia: Elsevier (2019). p. 699–712. 10.1016/B978-0-323-41509-5.00060-8

[2] Victor DW Sherman S Karakan T Khashab MA. Current endoscopic approach to indeterminate biliary strictures. World J Gastroenterol. (2012) 18:6197–205. 10.3748/wjg.v18.i43.6197 23180939 PMC3501767

[3] Kapoor BS Mauri G Lorenz JM. Management of biliary strictures: state-of-the-art review. Radiology. (2018) 289:590–603. 10.1148/radiol.2018172424 30351249

[4] Fidelman N. Benign biliary strictures: diagnostic evaluation and approaches to percutaneous treatment. Tech Vasc Interv Radiol. (2015) 18:210–7. 10.1053/j.tvir.2015.07.004 26615161

[5] Leng JJ Zhang N Dong JH. Percutaneous transhepatic and endoscopic biliary drainage for malignant biliary tract obstruction: a meta-analysis. World J Surg Oncol. (2014) 12:272. 10.1186/1477-7819-12-272 25148939 PMC6389255

[6] Banales JM Cardinale V Carpino G Marzioni M Andersen JB Invernizzi P. Expert consensus document: cholangiocarcinoma: current knowledge and future perspectives consensus statement from the European Network for the Study of Cholangiocarcinoma (ENS-CCA). Nat Rev Gastroenterol Hepatol. (2016) 13:261–80. 10.1038/nrgastro.2016.51 27095655

[7] DeOliveira ML Cunningham SC Cameron JL Kamangar F Winter JM Lillemoe KD. Cholangiocarcinoma: 31-year experience with 564 patients at a single institution. Ann Surg. (2007) 245:755–62. 10.1097/01.sla.0000251366.62632.d3 17457168 PMC1877058

[8] Nakeeb A Pitt HA Sohn TA Coleman J Abrams RA Piantadosi S. Cholangiocarcinoma. A spectrum of intrahepatic, perihilar, and distal tumors. Ann Surg. (1996) 224:463–73. 10.1097/00000658-199610000-00005 8857851 PMC1235406

[9] Razumilava N Gores GJ. Classification, diagnosis, and management of cholangiocarcinoma. Clin Gastroenterol Hepatol. (2013) 11:13–e4. 10.1016/j.cgh.2012.09.009 22982100 PMC3596004

[10] Blechacz B Komuta M Roskams T Gores GJ. Clinical diagnosis and staging of cholangiocarcinoma. Nat Rev Gastroenterol Hepatol. (2011) 8:512–22. 10.1038/nrgastro.2011.131 21808282 PMC3331791

[11] Bertuccio P Malvezzi M Carioli G Hashim D Boffetta P El-Serag HB. Global trends in mortality from intrahepatic and extrahepatic cholangiocarcinoma. J Hepatol. (2019) 71:104–14. 10.1016/j.jhep.2019.03.013 30910538

[12] Lindner P Rizell M Hafstrom L. The impact of changed strategies for patients with cholangiocarcinoma in this millenium. HPB Surg. (2015) 2015:736049. 10.1155/2015/736049 25788760 PMC4348584

[13] Kamsa-Ard S Luvira V Suwanrungruang K Kamsa-Ard S Luvira V Santong C. Cholangiocarcinoma trends, incidence, and relative survival in Khon Kaen, Thailand from 1989 through 2013: a population based cancer registry study. J Epidemiol. (2019) 29:197–204. 10.2188/jea.JE20180007 30078813 PMC6445798

[14] Dumonceau JM Delhaye M Charette N Farina A. Challenging biliary strictures: pathophysiological features, differential diagnosis, diagnostic algorithms, and new clinically relevant biomarkers-part 1. Ther Adv Gastroenterol. (2020) 13:1–27. 10.1177/1756284820927292 32595761 PMC7298429

[15] Yu XR Huang WY Zhang BY Li HQ Geng DY. Differentiation of infiltrative cholangiocarcinoma from benign common bile duct stricture using three-dimensional dynamic contrast-enhanced MRI with MRCP. Clin Radiol. (2014) 69:567–73. 10.1016/j.crad.2014.01.001 24581958

[16] Dorrell R Pawa S Zhou Y Lalwani N Pawa R. The diagnostic dilemma of malignant biliary strictures. Diagnostics. (2020) 10:337. 10.3390/diagnostics10050337 32466095 PMC7277979

[17] Lee HJ Cho KB. Diagnosis of malignant biliary stricture: more is better. Clin Endosc. (2018) 51:115–7. 10.5946/ce.2018.035 29618174 PMC5903087

[18] Joo I Lee JM Yoon JH. Imaging diagnosis of intrahepatic and perihilar cholangiocarcinoma: recent advances and challenges. Radiology. (2018) 288:7–13. 10.1148/radiol.2018171187 29869969

[19] Singh A Gelrud A Agarwal B. Biliary strictures: diagnostic considerations and approach. Gastroenterol Rep. (2015) 3:22–31. 10.1093/gastro/gou072 25355800 PMC4324869

[20] Yeo SJ Cho CM Jung MK Seo AN Bae HI. Comparison of the diagnostic performances of same-session endoscopic ultrasound-and endoscopic retrograde cholangiopancreatography-guided tissue sampling for suspected biliary strictures at different primary tumor sites. Korean J Gastroenterol. (2019) 73:213–8. 10.4166/kjg.2019.73.4.213 31030458 PMC12285762

[21] Shah RJ Langer DA Antillon MR Chen YK. Cholangioscopy and cholangioscopic forceps biopsy in patients with indeterminate pancreaticobiliary pathology. Clin Gastroenterol Hepatol. (2006) 4:219–25. 10.1016/S1542-3565(05)00979-1 16469683

[22] Alvaro D Bragazzi MC Benedetti A Fabris L Fava G Invernizzi P. Cholangiocarcinoma in Italy: a national survey on clinical characteristics, diagnostic modalities and treatment. Results from the “Cholangiocarcinoma” committee of the Italian association for the study of liver disease. Dig Liver Dis. (2011) 43:60–5. 10.1016/j.dld.2010.05.002 20580332

[23] Krishna N Tummala P Reddy AV Mehra M Agarwal B. Dilation of both pancreatic duct and the common bile duct on computed tomography and magnetic resonance imaging scans in patients with or without obstructive jaundice. Pancreas. (2012) 41:767–72. 10.1097/MPA.0b013e31823ba536 22450366

[24] Athanassiadou P Grapsa D. Value of endoscopic retrograde cholangiopancreatography-guided brushings in preoperative assessment of pancreaticobiliary strictures. Acta Cytol. (2011) 52:24–34. 10.1159/000325431 18323272

[25] Jailwala J Fogel EL Sherman S Gottlieb K Flueckiger J Bucksot LG. Triple-tissue sampling at ERCP in malignant biliary obstruction. Gastrointest Endosc. (2000) 51:383–90. 10.1016/S0016-5107(00)70435-4 10744806

[26] Farrell RJ Agarwal B Brandwein SL Underhill J Chuttani R Pleskow DK. Intraductal US is a useful adjunct to ERCP for distinguishing malignant from benign biliary strictures. Gastrointest Endosc. (2002) 56:681–7. 10.1067/mge.2002.128918 12397276

[27] Sun B Hu B. The role of intraductal ultrasonography in pancreatobiliary diseases. Endosc Ultrasound. (2016) 5:291–9. 10.4103/2303-9027.191607 27803901 PMC5070286

[28] Meister T Heinzow HS Woestmeyer C Lenz P Menzel J Kucharzik T. Intraductal ultrasound substantiates diagnostics of bile duct strictures of uncertain etiology. World J Gastroenterol. (2013) 19:874–81. 10.3748/wjg.v19.i6.874 23430958 PMC3574884

[29] Angsuwatcharakon P Kulpatcharapong S Moon JH Ramchandani M Lau J Isayama H. Consensus guidelines on the role of cholangioscopy to diagnose indeterminate biliary stricture. HPB. (2021) 24:17–29. 10.1016/j.hpb.2021.05.005 34172378

[30] Almadi MA Itoi T Moon JH Goenka MK Seo DW Rerknimitr R. Using single-operator cholangioscopy for endoscopic evaluation of indeterminate biliary strictures: results from a large multinational registry. Endoscopy. (2020) 52:574–82. 10.1055/a-1135-8980 32289852

[31] Krishna NB Saripalli S Safdar R Agarwal B. Intraductal US in evaluation of biliary strictures without a mass lesion on CT scan or magnetic resonance imaging: significance of focal wall thickening and extrinsic compression at the stricture site. Gastrointest Endosc. (2007) 66:90–6. 10.1016/j.gie.2006.10.020 17451708

[32] Dubow M Tatman PD Shah RJ. Individual probe based confocal laser endomicroscopy criteria in the analysis of indeterminate biliary strictures. Scand J Gastroenterol. (2018) 53:1358–63. 10.1080/00365521.2018.1512151 30394137

[33] Fugazza A Gaiani F Carra MC Brunetti F Lévy M Sobhani I. Confocal laser endomicroscopy in gastrointestinal and pancreatobiliary diseases: a systematic review and meta-analysis. Biomed Res Int. (2016) 2016:1–31. 10.1155/2016/4638683 26989684 PMC4773527

[34] Caillol F Bories E Autret A Poizat F Pesenti C Ewald J. Evaluation of pCLE in the bile duct: final results of EMID study: pCLE: impact in the management of bile duct strictures. Surg Endosc. (2015) 29:2661–8. 10.1007/s00464-014-3986-8 25492449

[35] Meining A Chen YK Pleskow D Stevens P Shah RJ Chuttani R. Direct visualization of indeterminate pancreaticobiliary strictures with probe-based confocal laser endomicroscopy: a multicenter experience. Gastroint Endosc. (2011) 74:961–8. 10.1016/j.gie.2011.05.009 21802675

[36] Taunk P Singh S Lichtenstein D Joshi V Gold J Sharma A. Improved classification of indeterminate biliary strictures by probe-based confocal laser endomicroscopy using the Paris Criteria following biliary stenting. J Gastroenterol Hepatol. (2017) 32:1778–83. 10.1111/jgh.13782 28294404

[37] Liew ZH Loh TJ Lim TKH Lim TH Khor CJ Mesenas SJ. Role of fluorescence in situ hybridization in diagnosing cholangiocarcinoma in indeterminate biliary strictures. J Gastroenterol Hepatol. (2018) 33:315–9. 10.1111/jgh.13824 28543841

[38] Brooks C Gausman V Kokoy-Mondragon C Munot K Amin SP Desai A. Role of fluorescent in situ hybridization, cholangioscopic biopsies, and EUS-FNA in the evaluation of biliary strictures. Dig Dis Sci. (2018) 63:636–44. 10.1007/s10620-018-4906-x 29353443

[39] Singhi AD Slivka A. Evaluation of indeterminate biliary strictures: is it time to FISH or cut bait? Gastrointest Endosc. (2016) 83:1236–8. 10.1016/j.gie.2016.02.002 27206587

[40] Martinez NS Trindade AJ Sejpal DV. Determining the indeterminate biliary stricture: cholangioscopy and beyond. Curr Gastroenterol Rep. (2020) 22:58. 10.1007/s11894-020-00797-9 33141356

[41] Bowlus CL Olson KA Gershwin ME. Evaluation of indeterminate biliary strictures. Nat Rev Gastroenterol Hepatol. (2016) 13:28–37. 10.1038/nrgastro.2015.182 26526122

[42] Helmy A Eldien HM Seifeldein GS Abu-Elfatth AM Mohammed AA. Digital image analysis has an additive beneficial role to conventional cytology in diagnosing the nature of biliary ducts stricture. J Clin Exp Hepatol. (2021) 11:209–18. 10.1016/j.jceh.2020.07.009 33746446 PMC7953004

